# Influenza A and B Viral Shedding in Adults Immunized With Live Attenuated Influenza Vaccine

**DOI:** 10.1093/cid/ciag294

**Published:** 2026-06-16

**Authors:** Brandon M Tower, Cindy M Liu, David J Diemert, Sydney G Nelson, Chelsea E Ware, Juan E Salazar, Tony Pham, Reed M Leventis, Katherine E Rose, Annie L S Roberts, Nathan O Weber, Edward H Sung, Maliha Aziz, Ryan M Troyer, Daniel E Park

**Affiliations:** Antibiotic Resistance Action Center, Department of Environmental and Occupational Health, Milken Institute School of Public Health, George Washington University, Washington, DC, USA; Antibiotic Resistance Action Center, Department of Environmental and Occupational Health, Milken Institute School of Public Health, George Washington University, Washington, DC, USA; George Washington University School of Medicine and Health Sciences, Department of Microbiology, Tropical Medicine and Immunology, Washington, DC, USA; Antibiotic Resistance Action Center, Department of Environmental and Occupational Health, Milken Institute School of Public Health, George Washington University, Washington, DC, USA; George Washington Vaccine Research Unit, George Washington Medical Faculty Associates, School of Medicine and Health Sciences, George Washington University, Washington, DC, USA; Antibiotic Resistance Action Center, Department of Environmental and Occupational Health, Milken Institute School of Public Health, George Washington University, Washington, DC, USA; Antibiotic Resistance Action Center, Department of Environmental and Occupational Health, Milken Institute School of Public Health, George Washington University, Washington, DC, USA; Antibiotic Resistance Action Center, Department of Environmental and Occupational Health, Milken Institute School of Public Health, George Washington University, Washington, DC, USA; Antibiotic Resistance Action Center, Department of Environmental and Occupational Health, Milken Institute School of Public Health, George Washington University, Washington, DC, USA; Antibiotic Resistance Action Center, Department of Environmental and Occupational Health, Milken Institute School of Public Health, George Washington University, Washington, DC, USA; Antibiotic Resistance Action Center, Department of Environmental and Occupational Health, Milken Institute School of Public Health, George Washington University, Washington, DC, USA; Antibiotic Resistance Action Center, Department of Environmental and Occupational Health, Milken Institute School of Public Health, George Washington University, Washington, DC, USA; Antibiotic Resistance Action Center, Department of Environmental and Occupational Health, Milken Institute School of Public Health, George Washington University, Washington, DC, USA; Department of Microbiology and Immunology, Schulich School of Medicine and Dentistry, Western University, London, ON, Canada; Antibiotic Resistance Action Center, Department of Environmental and Occupational Health, Milken Institute School of Public Health, George Washington University, Washington, DC, USA

**Keywords:** influenza, live attenuated influenza vaccine, viral shedding, immunization

## Abstract

**Background:**

Live attenuated influenza vaccine (LAIV) induces mucosal immunity through limited viral replication in the upper respiratory tract, but postvaccination viral shedding dynamics and their clinical correlates remain incompletely characterized.

**Methods:**

We evaluated 283 healthy adults (108 in 2023–2024, 175 during the 2024–2025 influenza seasons) following intranasal LAIV administration. Nasal swabs were collected on post-LAIV days 1, 2–4, and 5–7 to quantify influenza A and B RNA by RT-PCR. Viral detection, shedding duration and burden, clearance kinetics, and probability of detection were compared across seasons, vaccine strain compositions (quadrivalent vs trivalent), and host factors. Respiratory symptoms were assessed.

**Results:**

Early viral shedding was frequent: influenza A or B RNA was detected in 86.9% of participants on day 1, with codetection in 52.7%. Probability of detection declined from 92% on day 1% to 9% on day 7. Influenza B had longer shedding duration (median, 2.0 vs 1.0 days; *P* < .001) and higher shedding burden (4.2 vs 4.0 log_10_ RNA copies/mL; *P* < .001). Three clearance profiles—rapid (clearance ≤4 days), moderate (5–7 days), and slow (≥7 days)—were identified. Profiles were consistent across seasons but influenza B was disproportionately represented in slower-clearance groups (*P* < .001). Total viral burden (*P* < .01) and shedding duration (*P* = .01) were associated with total symptom burden.

**Conclusions:**

LAIV replication begins within 24 h and declines over the first week, with persistent shedding uncommon after day 7. Influenza B replicates more extensively and persists longer than influenza A. Symptom burden correlates with shedding duration and viral burden.

## Teaser

We report influenza A and B viral shedding in adults following live attenuated influenza vaccination. Influenza B exhibited greater replication and persistence, while prolonged shedding and higher viral burden were linked to increased respiratory symptoms and distinct viral shedding patterns.

Vaccination remains the most effective strategy for preventing severe influenza infections [1], which continue to cause substantial morbidity and mortality worldwide. The live attenuated influenza vaccine (LAIV) was first approved in 2003 in the United States for use in qualified individuals aged 5–49 years [2]. LAIV is administered intranasally and replicates in the upper respiratory tract, mimicking a natural infection, generating humoral and mucosal immune responses [3, 4]. Despite these advantages, LAIV efficacy has varied considerably across influenza seasons [5, 6], and is generally considered more immunogenic in pediatric populations [4, 7, 8]. This highlights the need to better understand host and vaccine factors that shape LAIV outcomes such as postvaccination viral shedding, which has been linked to the magnitude of immune responses [9, 10].

Most studies of post-LAIV shedding have focused on children [11–18], whereas influenza shedding dynamics in adults remains poorly defined [19–21]. Although adult studies have reported widely varying detection rates of influenza A and B following LAIV, they consistently report lower shedding rates in adults at 4.2%–50.0% [19–21], compared with the 8.9%–79.0% in pediatric populations [12–14]. Adult studies began sampling later than pediatric studies, suggesting early shedding could be an important part of adult LAIV response that was previously missed.

Vaccine composition may also contribute to the variability in seasonal post-LAIV shedding and vaccine efficacy. LAIV vaccine composition is generally updated annually, possibly resulting in adjustments in strain selection and the number of influenza A and B strains included. Prior studies in adolescent populations suggest vaccine composition can influence shedding patterns [5, 6, 16]; yet, its impact in adult populations is unknown. Understanding whether and how strain composition impacts postvaccination replication is critical for interpreting seasonal variations in LAIV performance and guiding future vaccine design.

To address these gaps, we conducted a study of healthy adults across 2 influenza seasons, where the 2023–2024 cohort received a quadrivalent LAIV and the 2024–2025 cohorts received a trivalent LAIV. We characterized the short-term dynamics of postvaccination influenza A and B viral RNA shedding starting on the first day post vaccination and assessed concurrent self-reported respiratory symptoms. By linking viral shedding dynamics to vaccine composition and symptom burden, this study provides new insights into LAIV replication in healthy adult populations.

## METHODS

### Study Design and Sample Collection

During the 2023–2024 and 2024–2025 influenza seasons, individuals aged 18–49 years who had elected to receive LAIV and had not yet received an influenza vaccine for the current season were recruited on and around George Washington University's Foggy Bottom campus and screened for eligibility. Exclusion criteria followed CDC LAIV guidelines and included systemic antibiotic or corticosteroid use in the previous 2 months; immune-related disorders requiring medication; or contraindications to anterior nasal sampling or venous blood draw. Participants that enrolled in the 2023–2024 cohort were not eligible for enrollment in the 2024–2025 cohort. Eligible participants were enrolled in our observational study.

In 2023–2024, participants received the quadrivalent FluMist® vaccine (AstraZeneca) containing ∼10^6.5–7.5^ fluorescent focus units of each of the 4 recombinant strains: A/Norway/31694/2022 (H1N1), A/Norway/16606/2021 (H3N2), B/Phuket/3073/2013 (B/Yamagata lineage), and B/Austria/1359417/2021 (B/Victoria lineage) [22]. In 2024–2025, participants received the trivalent FluMist® vaccine containing ∼10^6.5–7.5^ fluorescent focus units of each of the 3 recombinant strains: A/Norway/31694/2022 (H1N1), A/Perth/722/2024 (H3N2), and B/Austria/1359417/2021 (B/Victoria lineage) [23]. Study screening form and participant questionnaire can be found in Supplementary Table 1. A total of 7 participants were excluded due to missing 2 or more follow-up visits.

Participants self-collected nasal swabs at the baseline visit prior to receiving LAIV and at 3 additional post-LAIV visits on day 1, day 2–4, and day 5–7. At each study visit, patients completed the Wisconsin Upper Respiratory Symptom Survey (WURSS-11) [24]. Swabs were immediately collected into 1 mL of DNA/RNA shield (R1100-250, Zymo), stored at 4°C, and processed within 2 h. All participants provided written informed consent. The study protocol was approved by the George Washington University Institutional Review Board (NCR234806).

### Influenza A and B Detection and Quantification

RNA was extracted from 50 µL of swab eluent using the MagMAX™-96 Viral RNA Isolation Kit (Thermo Fisher) using a final elution volume of 50 µL. Influenza A and B RNA were quantified by RT-qPCR adapted from Boikos et al [14], with human ribonuclease P (RP) as an internal extraction control (Supplementary Figure 1). Reactions were performed using the QuantiTect One-Step RT-PCR Kit (Qiagen) on the LightCycler® 480 II (Roche). Each run included plasmid standards, negative template, and negative subject controls. Limits of detection and assay validation procedures are described in the Supplementary Methods.

### Data Analysis

The primary endpoints were post-LAIV influenza viral detection, duration of viral shedding, and postvaccination symptoms. Influenza A and B detection and shedding were measured based on RT-qPCR cycle threshold (Ct) values converted to virus copy numbers using in-run standard curves. Continuous variables are presented as medians with interquartile range (IQR), and categorical variables are expressed as frequencies. Proportions and 95% CIs were estimated using a binomial distribution.

Proportions were compared using the two-proportion z-test, *χ*^2^ test, or Fisher exact test, as appropriate, and medians were compared using the Mann–Whitney *U* test or Kruskal–Wallis test. Predicted probabilities and 95% CIs of influenza detection were estimated using logistic mixed-effects regression models to account for repeated measures. Logistic regression models were used to assess associations between host factors, influenza season, and detection of influenza. Duration of viral shedding was analyzed using Cox proportional hazards models, with results reported as hazard ratios (HRs) and 95% CIs. Linear mixed-effects regression models were used to estimate the viral load decay rate. Interaction terms between the slope (days postvaccination) and influenza type or codetection status were included to assess whether viral load decay differed by influenza type or codetection status.

Total symptom burden and total viral burden were determined by calculating the area under the curve (AUC) of reported WURSS-11 symptom sum score and viral load over the follow-up period using the trapezoidal rule (Supplementary Figure 2). Linear regression models were used to assess predictors that are associated with total symptom burden. All statistical analyses were conducted in SAS version 9.4, and all figures and graphs were prepared in R version 4.4.2.

## RESULTS

### Study Population

A total of 283 participants (108 in 2023–2024 and 175 in 2024–2025) were included in our analysis. Participants had a median age of 22 years (IQR: 20–26 and range: 18–49) and were predominantly female (71.4%) and Caucasian (64.7%) (Table 1). Most (62.5%) participants had received influenza vaccination within 14 months prior to their enrollment date (Table 1). Recent smoking was the only participant characteristic to differ by influenza season (Supplementary Table 2). In the 2023–2024 season, 94.6%, 97.3%, and 92.8% participants completed post-LAIV day 1, day 2–4, and day 5–7 visits, respectively. In the 2024–2025 season, 96.6%, 97.2%, 96.1% participants completed post-LAIV day 1, day 2–4, and day 5–7 visits, respectively. The distribution of RP Ct values did not differ by study visit (Supplementary Figure 1).

**Table 1.** Baseline Characteristics of Study Participants

Characteristic	No. (%)
Sex	
Female	202 (71.4%)
Male	81 (28.6%)
Age, years	
Age, median (Q1-Q3)	23 (20–26)
Range	18–49
Race	
Caucasian	183 (64.7%)
Black or African American	31 (11.0%)
Asian	47 (16.6%)
Multiracial	13 (4.6%)
Missing	9 (3.2%)
Ethnicity	
Hispanic or Latino	39 (13.8%)
Not Hispanic or Latino	244 (86.2%)
Influenza vaccination^a^	
Yes	177 (62.5%)
No	105 (37.1%)
Missing	1 (0.4%)
Recent smoking^b^	
Yes	51 (18.0%)
No	232 (82.0%)
Environmental allergies	
Yes	141 (49.8%)
No	141 (49.8%)
Missing	1 (0.4%)

^a^Defined as receipt of an influenza vaccine within 14 m prior to their enrollment.

^b^Defined as use of a cigarette, e-cigarette, cigar, or hookah within 30 days of enrollment.

### Influenza A and B Detection Following Live Attenuated Influenza Vaccination

Within 1 week of LAIV vaccination, influenza A and B viral RNAs were frequently detected in participants’ nasal cavities. Influenza A was detected in 172 (60.8%) participants, and influenza B in 242 (85.6%) participants (Figure 1*A*); overall, 258 (91.2%) participants had either influenza A or B detected within the first week (Figure 1*B*). Codetection of influenza A and B occurred in 156 (55.1%) participants, whereas 16 (5.7%) had influenza A only and 86 (30.4%) had influenza B only (Figure 1*B*).

**Figure 1.**
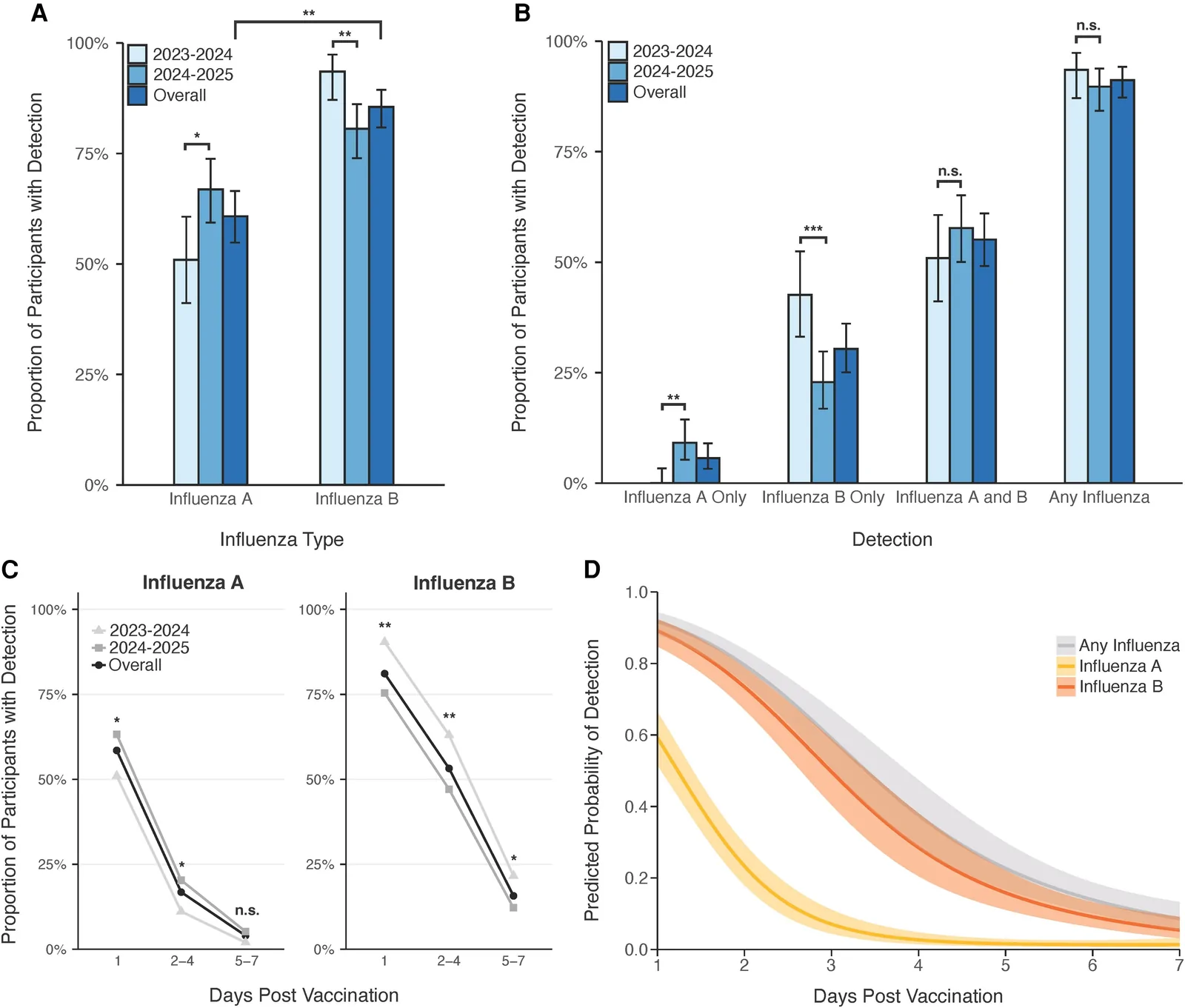
Detection rates and probability of detection of influenza A and B following live attenuated influenza vaccination. *A*, Overall rate of RT-qPCR-confirmed influenza B detection (86.6%) was significantly higher than influenza A detection (61.5%) (*P* < .001). Influenza A detection was significantly higher in the 2024–2025 season compared with the 2023–2024 season (66.9% vs 50.9%, *P* < .05). Influenza B detection was significantly higher in the 2023–2024 season compared with the 2024–2025 season (93.5% vs 80.6%, *P* < .01). *B*, Seasonal differences were observed for influenza A only and influenza B only, while codetection (influenza A and B) and overall influenza detection did not differ significantly by season. *C*, Influenza B was detected at higher rates than influenza A at all postvaccination visits. Influenza A was detected at higher rates at post-LAIV day 1 and day 2–4 in the 2024–2025 seasons. Influenza B was detected at higher rates at all study time points in the 2023–2024 season. *D*, Predicted probabilities from logistic mixed-effects regression models, estimated among all participants, show distinct detection probabilities for influenza A and B. The probability of influenza A detection declined more rapidly, whereas the probability of influenza B detection remained higher than influenza A and very similar to the probability of any influenza detection. Shaded regions represent 95% CIs. All statistical tests comparing proportions used the two-proportion z-test. Asterisks represent significant *P* values (* *P* < .05, ** *P* < .01, *** *P* < .001).

Post-LAIV viral detection patterns varied by year of administration. With the quadrivalent vaccine (2 influenza A and 2 influenza B strains) used in the 2023–2024 season, influenza B detection was more common compared with the 2024–2025 season (93.5% vs 80.6%, *P* < .01, Figure 1*A*). In contrast, with the trivalent vaccine (2 influenza A strains and 1 influenza B strain), influenza A detection was more common compared with the 2023–2024 season (66.9% vs 50.9%, *P* = .01, Figure 1*A*). Age, sex, prior influenza vaccination, and recent smoking status were not significantly associated with post-LAIV influenza detection in either season (Table 2; Supplementary Table 3). These findings suggest that changes in vaccine strain composition across years may impact post-LAIV viral detection.

**Table 2.** Association of Influenza A and B Detection With Influenza Season and Host Factors

	Influenza A			Influenza B		
Variable	n/N (%)	aOR^a^ (95% CI)	*P* value	n/N (%)	aOR^a^ (95% CI)	*P* value
Influenza season						
2023–2024	55/108 (50.9%)	0.51 (0.31, 0.84)	**0.01**	101/108 (93.5%)	3.38 (1.43, 8.00)	**.01**
2024–2025	117/175 (66.9%)	Ref		141/175 (80.6%)	Ref	
Influenza vaccination						
Yes	106/177 (59.9%)	0.90 (0.54, 1.50)	0.68	152/177 (85.9%)	0.99 (0.49, 2.02)	.98
No	66/105 (62.9%)	Ref		89/105 (84.8%)	Ref	
Recent smoking						
Yes	33/51 (64.7%)	1.09 (0.56, 2.10)	0.81	40/51 (78.4%)	0.67 (0.30, 1.51)	.34
No	139/232 (59.9%)	Ref		202/232 (87.1%)	Ref	
Sex						
Female	126/202 (62.4%)	1.27 (0.74, 2.19)	0.39	174/202 (86.1%)	1.22 (0.58, 2.57)	.60
Male	46/81 (56.8%)	Ref		68/81(84.0%)	Ref	
Age		1.00 (0.96, 1.05)	0.83		1.02 (0.96, 1.08)	.58

Bold *P* values represent statistical significance at the *P* < .05 level.

Abbreviations: aOR, adjusted odds ratio; CI, confidence interval.

^a^Multivariate logistic regression adjusted for all variables in table.

### Duration and Burden of Influenza A and B Viral Shedding Following Live Attenuated Influenza Vaccination

The duration and burden of viral shedding following LAIV differed significantly between influenza A and B. Shedding duration was longer for influenza B than influenza A (median, 2.0 vs 1.0 days; *P* < .001, Supplementary Table 4). Influenza B also demonstrated a higher overall shedding burden than influenza A throughout the study period (*P* < .001), with a median of 4.2 log_10_ RNA copies per swab on post-LAIV day 1 compared with 4.0 log_10_ RNA copies for influenza A (Supplementary Table 4).

Peak viral shedding occurred on post-LAIV day 1 for both virus types, with 82.0% and 72.6% of participants exhibiting peak influenza A and B shedding, respectively. By the post-LAIV day 2–4 visit, influenza A was detected in only 16.8% of participants, whereas influenza B remained detectable in 53.2% of participants. By the post-LAIV day 5–7 visit, influenza A detection decreased to 4.0%, while influenza B remained detectable in 15.7% of participants (Figure 1*C*). These findings demonstrate greater replication and persistence of influenza B in the upper respiratory tract than influenza A following LAIV.

Post-LAIV viral shedding patterns varied by influenza season. During the 2023–2024 season (quadrivalent vaccine), influenza A and influenza B exhibited a higher peak viral shedding burden than the 2024–2025 season (*P* < .001, Supplementary Table 4). Viral shedding was higher with the quadrivalent vaccine for both influenza A and B, but this difference was only observed on post-LAIV day 1. The total viral shedding burden was also higher for influenza B when a quadrivalent vaccine was used (*P* < .001, Supplementary Table 4), but did not differ for influenza A. These findings suggest that changes in vaccine strain composition across years may impact other viral shedding outcomes such as peak and total viral shedding burden post-LAIV.

### Influenza A and B Viral Clearance Following Live Attenuated Influenza Vaccination

Viral clearance rate differed significantly between influenza A and B, with a 62% faster clearance rate for influenza A compared with influenza B (adjusted HR 1.62, 95% CI 1.29–2.03, Supplementary Table 5). This difference was reflected in the distribution of 3 distinct viral clearance phenotypes: slow (viral shedding for ≥7 days post-LAIV), moderate (viral shedding through 2–4 days post-LAIV, clearance by day 5–7), and rapid (clearance within 2–4 days post-LAIV). Total viral burden varied significantly by viral clearance profile (median = 3.74 for rapid; 14.5 for moderate; 22.6 for low). Age, sex, prior influenza vaccination, and recent smoking status were not associated with viral clearance profiles (Supplementary Table 6).

The distribution of viral clearance profiles differed by influenza type (*χ*^2^  *P* < .001, Supplementary Table 4). Influenza B clearance profiles were more likely to be slow (15.7% vs 5.0%, *P* = .001) and moderate (47.5% vs 20.6%, *P* < .001) compared with influenza A and less likely to be rapid (36.8% vs 74.4%, *P* < .001). Viral decay rates differed across the 3 viral clearance profiles (slow vs moderate vs fast) but did not differ by influenza type or codetection status, suggesting that decay patterns align closely with clearance phenotype classification (Figure 2; Supplementary Table 7). In contrast to the significant variations in detection rates and peak viral shedding burden between 2023–2024 and 2024–2025 seasons, viral shedding duration and the distribution of viral clearance profiles for influenza A and influenza B were consistent across years (Supplementary Table 4).

**Figure 2.**
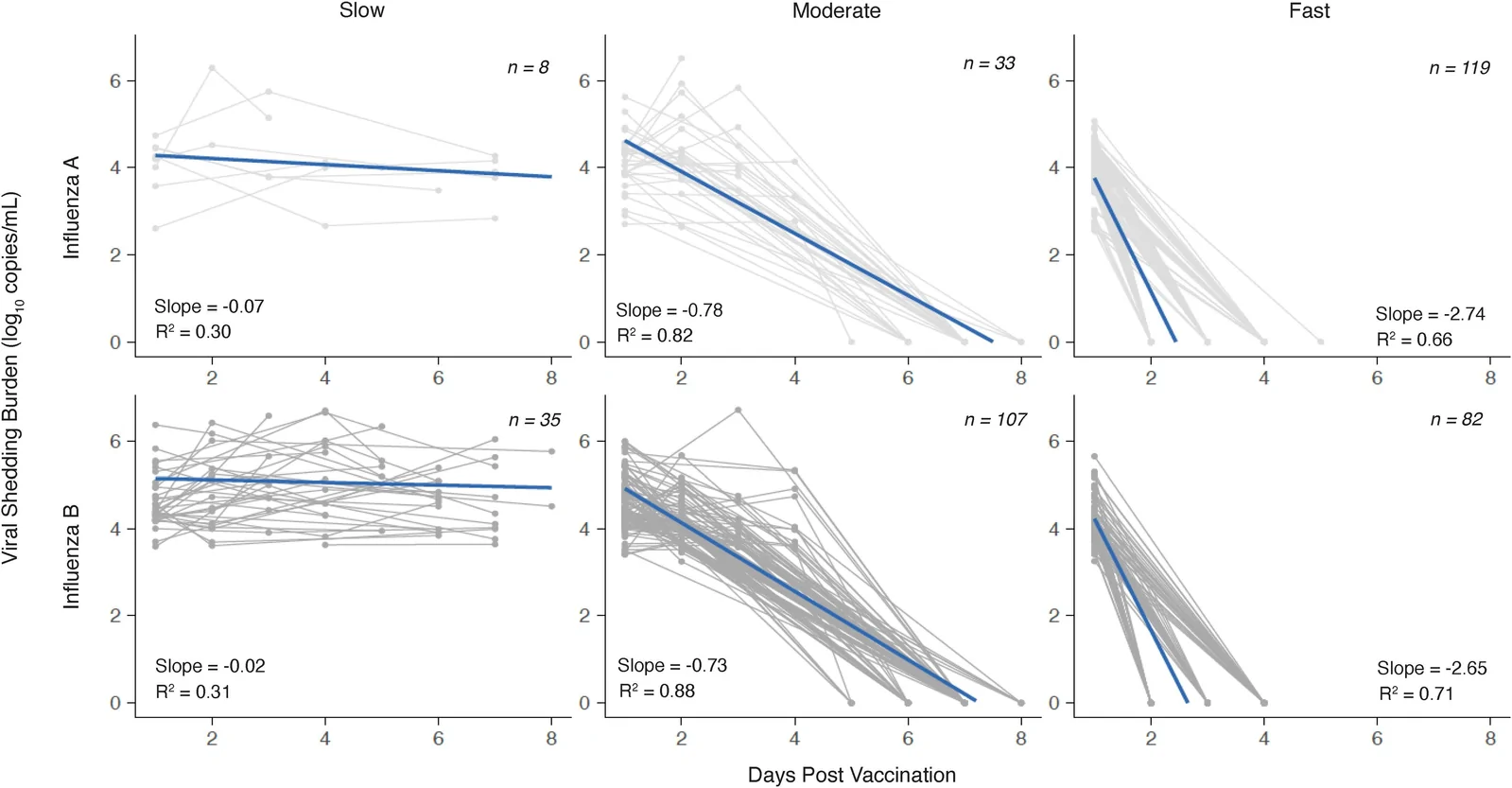
Viral clearance profiles of influenza A and B following live attenuated influenza vaccination. Spaghetti plots show viral shedding over 3 postvaccination intervals (post-LAIV day 1, day 2–4, and day 5–7) in participants with RT-qPCR-confirmed influenza A or B, stratified by influenza type and viral clearance profile. Gray lines represent individual participants, and blue lines indicate group-level trends. Slope and conditional *R*^2^ for each linear mixed-effect model are reported in black text. Sample size of each viral clearance profile is reported in black text.

### Probability of Viral Detection by Day Following Live Attenuated Influenza Vaccination

To characterize the temporal dynamics of post-LAIV viral replication, we modeled the probability of influenza RNA detection by day following vaccination. Model-based estimates showed a rapid decline in detection probability after vaccination. The predicted probability of detecting influenza A or B RNA was 92% (95% CI, 88%–94%) on day 1, 23% (17%–30%) on day 5, and 9% (5%–13%) by day 7 (Table 3). Influenza B had higher day-1 predicted detection (89% vs 59% for influenza A) and a slower decline across time points (Figure 1*D*). These findings indicate that viral replication begins promptly after vaccination but decreases sharply thereafter, with most adults clearing detectable RNA within the first week.

**Table 3.** Predicted Probability of Influenza Detection by Day Postvaccination

	Influenza A	Influenza B	Any Influenza
Day Postvaccination	Mean Predicted Probability (95% CI)^a^	Mean Predicted Probability (95% CI)^a^	Mean Predicted Probability (95% CI)^a^
1	0.59 (0.52, 0.66)	0.89 (0.85, 0.92)	0.92 (0.88, 0.94)
2	0.23 (0.18, 0.30)	0.73 (0.67, 0.79)	0.80 (0.74, 0.84)
3	0.07 (0.04, 0.11)	0.50 (0.40, 0.59)	0.59 (0.51, 0.67)
4	0.03 (0.01, 0.05)	0.28 (0.21, 0.38)	0.38 (0.29, 0.47)
5	0.02 (0.01, 0.03)	0.16 (0.11, 0.22)	0.23 (0.17, 0.30)
6	0.01 (0.01, 0.03)	0.09 (0.06, 0.13)	0.14 (0.10, 0.19)
7	0.01 (0.01, 0.03)	0.05 (0.03, 0.09)	0.09 (0.05, 0.13)

Abbreviation: CI, confidence interval.

^a^Predicted probabilities are model-based estimates from a logistic mixed-effects regression model, and represent predicted probabilities for all study participants.

### Association Between Viral Shedding and Respiratory Symptoms Following Live Attenuated Influenza Vaccination

Mild respiratory symptoms were common during post-LAIV follow-up visits. The most common symptoms were runny nose (58.3%), feeling tired (58.0%), and nasal congestion (50.2%), while sore throat (27.9%), scratchy throat (24.0%), and itchy nose (20.8%) were the least common (Supplementary Table 8). The median total symptom burden at the baseline vaccination visit was 1.0 (Q1: 0.0; Q3: 3.0). Symptom patterns varied across visits, with feeling tired (40.7%) being the most common on post-LAIV day 1, runny nose (41.8%) being the most common during days 2–4 and feeling tired (33.5%) again the most common during days 5–7 (Supplementary Table 8). The median total symptom burden was 12.8 (Q1: 5.0; Q3: 30.8), with median scores of 17.2 and 15.0 for those with influenza A, and influenza B detected, respectively. Symptom burden differed for those with and without virus detected (*P* < .01, Supplementary Figure 3). Symptom burden was higher among those with influenza A detected compared with influenza B (Supplementary Figure 4). Total symptom burden did not differ between participants with detectable influenza A or B across influenza seasons (*P* = .42, Supplementary Table 9).

Among the viral shedding parameters, total viral shedding burden and total duration of viral shedding were the strongest predictors of total symptom burden (Table 4). Although influenza A exhibited a shorter duration of viral shedding and lower total viral burden than influenza B (Supplementary Table 4 and Supplementary Figure 5), its shedding duration was more strongly associated with symptom burden (Supplementary Table 10). These findings indicate that greater viral replication and prolonged shedding contribute to increased respiratory symptom burden following LAIV administration, and that influenza A strains may have a greater impact on symptom burden.

**Table 4.** Viral Determinants of Respiratory Symptoms

Variable	Estimate^a^ (95% CI)	*P* Value
Total viral burden^b^	0.42 (0.15, 0.70)	.**003**
Total duration of viral^b^ shedding	1.65 (0.39, 2.91)	.**01**
Total peak viral load^b^	0.41 (−0.56, 1.38)	.41
Influenza A clearance profile		
Fast	−8.19 (−18.11, 1.72)	.10
Slow	1.97 (−17.88, 21.82)	.84
Moderate	Ref	
Influenza B clearance profile		
Fast	−4.10 (−11.45, 3.25)	.27
Slow	−1.10 (−10.83, 8.64)	.82
Moderate	Ref	

Bold *P* values represent statistical significance at the *P* < .05 level.

Abbreviations: CI, confidence interval.

^a^Multivariate linear regression adjusting for age, sex, influenza vaccination, and environmental allergies.

^b^Total viral determinants were calculated by summing total viral burden (AUC) for influenza A and B for each participant.

## DISCUSSION

To our knowledge, this is the first study to characterize early post-LAIV shedding in an adult population. We found that post-LAIV viral shedding in adults may be comparable to those observed in pediatric populations. Specifically, 91% of adults in our study had detectable influenza A or B RNA after LAIV, in contrast to prior reports showing 4.2%–50.0% detection of influenza viral RNA in adults [19–21]. This likely reflects earlier sampling, short shedding duration, differences in vaccine strain composition [5, 6, 16], and more sensitive RT-qPCR detection [25] compared with immunofluorescence or tissue culture used in earlier studies [19–21]. Given that post-LAIV shedding has been linked to the magnitude of immune responses [9, 10], the higher shedding rate we observed in adults may provide a biological explanation for why LAIV efficacy in adults can be similar to that observed in children.

Vaccine composition may impact postvaccination viral RNA detection and shedding patterns. Reducing influenza B strains from 2 (2023–2024) to 1 (2024–2025) coincided with higher rates of influenza A shedding and lower rates of influenza B shedding. Because early mucosal viral dynamics have been shown to influence downstream systemic antibody responses [10], differences in replication driven by strain composition may have immunologic relevance. Further studies are needed to determine how vaccine composition impacts post-LAIV shedding and immunity, and how these outcomes interact with baseline pre-existing immunity.

We also observed that post-LAIV viral shedding was associated with symptom burden in adults, similar to what has been reported in children and adults with RT-qPCR-confirmed influenza infections [26, 27]. Influenza A typically causes more severe illness than influenza B among children and adolescents [28], which may also explain why we observed a significant association between symptom burden and influenza A shedding patterns, but not influenza B.

Consistent with earlier studies [15, 16, 19, 21], influenza B was detected more frequently than influenza A after LAIV and was associated with more persistent and higher levels of viral shedding. By combining frequency, duration, and shedding levels, we observed 3 distinct viral patterns—slow, moderate, and rapid—that were applicable to both influenza types. Influenza A was predominantly associated with the rapid clearance profile, while influenza B was more frequently associated with moderate and slow clearance profiles. These patterns likely reflect the presence of coordinated nasal immune programs that include contributions from local and systemic pre-existing and innate immunity. Community influenza circulation in preceding years may further modulate these responses; for example, the overwhelming predominance of influenza A in the 2021–2022 and 2022–2023 US seasons could have increased baseline anti-A immunity and suppressed LAIV-A replication.

Our model-based estimates of the probability of viral RNA detection by day post-LAIV support that detection was highly likely at day 1 (92%) but declined steeply over time, reaching 23% by day 5% and 9% by day 7. These dynamics reinforce that LAIV replication begins rapidly after vaccination but is short-lived in most adults. Although clinical transmission from vaccine recipients has not been demonstrated in real-world settings and ACIP considers the risk of onward transmission to be theoretical and relevant primarily to close contacts of severely immunocompromised individuals, the rapid decrease in detection probability we observed suggests that, even if secondary transmission were possible, opportunities would be largely restricted to the earliest days following vaccination [29].

Our study had several limitations. Our study population was composed primarily of younger, white, and female adults, which could limit the generalizability of our study. We also have not characterized baseline immune status of our study population yet, which has been shown to influence post-LAIV shedding [20, 30, 31]. This limitation is to be addressed in further studies, as characterization of baseline immune status is underway. Additionally, while RT-qPCR is routinely used as a clinical diagnostic for influenza and to assess LAIV nasal shedding postvaccination in research studies [14–17, 26], PCR-based methods cannot specifically discriminate between infectious virions and noninfectious viral nucleic acids. This ultimately limits our ability to draw specific conclusions related to contagiousness of LAIV vaccines. Further, early detection may partially reflect residual vaccine virus; nonetheless, the subsequent shedding kinetics and subtype-specific patterns indicate active replication rather than passive persistence alone. Despite these limitations, our findings provide the most detailed characterization to date of early post-LAIV shedding in adults, which can have important implications for our understanding of the mucosal vaccine efficacy in the adult population.

In summary, we found that post-LAIV viral shedding in a healthy adult population is substantially more common than previously reported and is similar to rates in pediatric populations. In particular, influenza A shedding was a significant determinant of post-LAIV respiratory symptoms. Further studies are needed to understand how post-LAIV viral shedding dynamics, including the 3 distinct viral shedding profiles observed in this study, impact LAIV efficacy, and to determine vaccine compositions that best balance viral shedding, symptom burden, transmission risk, and protective immunogenicity.

## Supplementary Material

ciag294_Supplementary_Data

## Supplementary Data

Supplementary materials are available at *Clinical Infectious Diseases* online. Consisting of data provided by the authors to benefit the reader, the posted materials are not copyedited and are the sole responsibility of the authors, so questions or comments should be addressed to the corresponding author.

## Notes

***Acknowledgments***. We would like to thank all the participants of our study that generously volunteered their time and effort. We would also like to thank Megan Spyers-Duran, Jade Ditta, Skylar Adderley, Lauren Knight, Marwa Hameed, Isabela Burton, and Stephanie Metis for their time, effort, and support with sample processing. The successful completion of this publication would not have been possible without the contributions of all participants and lab staff.

***Data availability***. The data underlying this article will be shared on reasonable request to the corresponding author.

***Financial support*.** This work was supported by the National Institutes of Health (grant number 5R01AI168182-03) and a Research Enhancement Award from the Office of Research Excellence, Milken Institute School of Public Health, George Washington University, USA.
