# Supplementary material for: Influenza A and B Viral Shedding in Adults Immunized With Live Attenuated Influenza Vaccine
Source: Clin Infect Dis. 2026 Jun 16;83(3):e611–8. doi: 10.1093/cid/ciag294 (PMC13644873; doi:10.1093/cid/ciag294)
Supplement: Supplementary file 1 [file ciag294_supplementary_data.docx]

**Supplementary Material**

**Supplement To:** “Influenza A and B Shedding in Adults Immunized With Live-Attenuated Influenza Vaccine”

**Table S1. Study participant screening form and follow-up visit questionnaire**

| **Screening Form Question** | **Possible Answers** |
| --- | --- |
| Would you be able to attend regular study visits at the GW campus in Foggy Bottom, Washington DC?A total of five in-person study visits (one 30-minute enrollment visit, three 10-minute followup visits during the first week, 1 20-minute visit after 6 weeks) will be conducted on the GW campus in Foggy Bottom, Washington, DC. | Yes  No |
| Are you between 18 and 49 years of age? | Yes  No |
| What is your age (years)? | Text (integer) |
| Have you previously participated in our FluMist study? | Yes  No |
| Do you have an allergy to an ingredient of the influenza vaccine? | Yes  No  Don’t know |
| Have you ever had a serious reaction to influenza vaccine in the past? | Yes  No  Don’t know |
| Do you have an open channel between the cerebrospinal fluid (CSF) and the mouth, throat, nose, or ear or any other cranial CSF leak? | Yes  No  Don’t know |
| Do you have a cochlear implant? | Yes  No  Don’t know |
| Are you pregnant or could you become pregnant within the next month? | Yes  No Don’t know |
| Within 4 weeks before or after LAIV administration, will you receive Yellow fever, MMR, Varicella, or other live vaccines? | Yes  No  Don’t know |
| Are you currently on antibiotics, or have you taken antibiotics within the last two months? | Yes  No |
| Have you received a flu shot (intramuscular) this current flu season? | Yes  No |
| **Follow-Up Visit Questionnaire** | **Possible Answers** |
| Have you taken any antibiotics since your last visit? | Yes  No |
| How sick do you feel today? | Yes  No |
| Please rate the average severity of the following symptoms over the last 24 hours:  Runny Nose  Plugged Nose  Sneezing  Sore Throat  Scratchy Throat  Cough  Feeling Tired  Itchy Nose  Other | 0 – Have not had this symptom  1 – Very Mildly  2  3 – Mildly  4  5 – moderately  6  7 - Severe |

**Table S2. Baseline characteristics of study participants by influenza season**

| **Characteristic** | **Total (n=283),**  **No. (%)** | **2023-2024 (n=108),**  **No. (%)** | **2024-2025 (n=175),**  **No. (%)** | **p-value^c^** |
| --- | --- | --- | --- | --- |
| **Sex** |  |  |  | 0.70 |
| Female | 202 (71.4%) | 79 (73.1%) | 123 (70.3%) |  |
| Male | 81 (28.6%) | 29 (26.9%) | 52 (29.7%) |  |
| **Age, years** |  |  |  | 0.42 |
| Age**,** median (Q1-Q3) | 23 (20-26) | 22 (20-26) | 23 (20.5-27) |  |
| Range | 18 - 49 | 18-46 | 18-49 |  |
| **Race** |  |  |  | 0.39 |
| Caucasian | 183 (64.7%) | 76 (70.4%) | 107 (61.1%) |  |
| Black or African American | 31 (11.0%) | 9 (8.3%) | 22 (12.6%) |  |
| Asian | 47 (16.6%) | 18 (16.7%) | 29 (16.6%) |  |
| Multiracial | 13 (4.6%) | 3 (2.8%) | 10 (5.7%) |  |
| Missing | 9 (3.2%) | 2 (1.9%) | 7 (4.0%) |  |
| **Ethnicity** |  |  |  | 0.89 |
| Hispanic or Latino | 39 (13.8%) | 14 (13.0%) | 25 (14.3%) |  |
| Not Hispanic or Latino | 244 (86.2%) | 94 (87.0%) | 150 (85.7%) |  |
| **Influenza Vaccination^a^** |  |  |  | 0.86 |
| Yes | 177 (62.5%) | 69 (63.9%) | 108 (61.7%) |  |
| No | 105 (37.1%) | 39 (36.1%) | 66 (37.7%) |  |
| Missing | 1 (0.4%) | 0 (0.0%) | 1 (0.6%) |  |
| **Recent Smoking^b^** |  |  |  | **0.03** |
| Yes | 51 (18.0%) | 12 (11.1%) | 39 (22.3%) |  |
| No | 232 (82.0%) | 96 (88.9%) | 136 (77.7%) |  |
| **Environmental Allergies** |  |  |  | 0.54 |
| Yes | 141 (49.8%) | 51 (47.2%) | 90 (51.4%) |  |
| No | 141 (49.8%) | 57 (52.8%) | 84 (48.0%) |  |
| Missing | 1 (0.4%) |  |  |  |
|  |  |  |  |  |

Bold p-values represent statistical significance at the p < 0.05 level

**^a^** Defined as receipt of an influenza vaccine within 14 months prior to their enrollment

^b^ Defined as use of a cigarette, e-cigarette, cigar, or hookah within 30 days of enrollment

^c^ For continuous variables, the Mann Whitney U-test was used, and for categorical variables the Chi-Squared test was used when all cell counts > 5, and Fisher’s exact test when any cell count < 5

**Table S3. Association of influenza outcomes with influenza season and host factors**

|  | **Only Influenza A** | | |  | **Only Influenza B** | | |  | **Influenza A and B** | | |  | **Any Influenza** | | |
| --- | --- | --- | --- | --- | --- | --- | --- | --- | --- | --- | --- | --- | --- | --- | --- |
| **Variable** | **n/N (%)** | **aOR^b^**  **(95% CI)** | **p-value** |  | **n/N (%)** | **aOR^b^**  **(95% CI)** | **p-value** |  | **n/N (%)** | **aOR^b^**  **(95% CI)** | **p-value** |  | **n/N (%)** | **aOR^b^**  **(95% CI)** | **p-value** |
| **Influenza Season** |  |  |  |  |  |  |  |  |  |  |  |  |  |  |  |
| 2023-2024 | 0/108  (0.0%) | N/A^a^ |  |  | 46/108  (42.6%) | 2.58  (1.52, 4.38) | **<0.001** |  | 55/108  (50.9%) | 0.73  (0.48, 1.20) | 0.21 |  | 101/108  (93.5%) | 1.71  (0.68, 4.30) | 0.25 |
| 2024-2025 | 16/175  (9.1%) | Ref | - |  | 40/175  (22.9%) | Ref | - |  | 101/175  (57.7%) | Ref | - |  | 157/175  (89.7%) | Ref | - |
| **Influenza Vaccination** |  |  |  |  |  |  |  |  |  |  |  |  |  |  |  |
| Yes | 9/177  (5.1%) | N/A^a^ |  |  | 55/177  (31.1%) | 1.11  (0.64, 1.93) | 0.70 |  | 97/177  (54.8%) | 0.91  (0.56, 1.51) | 0.73 |  | 161/177  (91.0%) | 0.96  (0.40, 2.31) | 0.93 |
| No | 7/105  (6.7%) | Ref | - |  | 30/105  (28.6%) | Ref | - |  | 59/105  (56.2%) | Ref | - |  | 96/105  (91.4%) | Ref | - |
| **Recent Smoking** |  |  |  |  |  |  |  |  |  |  |  |  |  |  |  |
| Yes | 7/51  (13.7%) | N/A^a^ |  |  | 14/51  (27.5%) | 1.03  (0.50, 2.09) | 0.95 |  | 47/51  (92.2%) | 0.78  (0.41, 1.46) | 0.43 |  | 47/51  (92.2%) | 1.33  (0.42, 4.19) | 0.63 |
| No | 9/232  (3.9%) | Ref | - |  | 72/232  (31.0%) | Ref | - |  | 211/232  (90.9%) | Ref | - |  | 211/232  (90.9%) | Ref | - |
| **Sex** |  |  |  |  |  |  |  |  |  |  |  |  |  |  |  |
| Female | 12/202  (5.9%) | N/A^a^ |  |  | 60/202  (29.7%) | 0.90  (0.50, 1.62) | 0.73 |  | 114/202  (56.4%) | 1.19  (0.70, 2.03) | 0.52 |  | 186/202  (92.1%) | 1.50  (0.62, 3.62) | 0.37 |
| Male | 4/81  (4.9%) | Ref | - |  | 26/81  (32.1%) | Ref | - |  | 42/81  (51.9%) | Ref | - |  | 72/81  (88.9%) | Ref | - |
| **Age^a^** | - |  |  |  | - | 0.99  (0.63, 1.54) | 0.96 |  | - | 1.10  (0.73, 1.64) | 0.65 |  | - | 1.10  (0.55, 2.19) | 0.78 |
|  |  |  |  |  |  |  |  |  |  |  |  |  |  |  |  |

Abbreviations: aOR, adjusted odds ratio, CI, confidence interval.

Bold p-values represent statistical significance at the p < 0.05 level

**^a^** Logistic regression model did not converge

^b^ Multivariate logistic regression adjusted for all variables in table

**Table S4. Summary of viral shedding outcomes and viral clearance profiles by influenza type and influenza season**

|  | **Influenza A** | | |  | **Influenza B** | | |  |  |  |  |
| --- | --- | --- | --- | --- | --- | --- | --- | --- | --- | --- | --- |
| **Viral Shedding Outcome^a^** | **A. Overall (n=283)** | **B. 2023-2024 (n=108)** | **C. 2024-2025 (n=175)** |  | **D. Overall**  **(n = 283)** | **E. 2023-2024 (n=108)** | **F. 2024-2025 (n=175)** |  | **A v D**  **p-value** | **B v C**  **p-value** | **E v F**  **p-value** |
| **Duration of Shedding** (days), median (Q1-Q3) | 1.0 (1.0-2.0) | 1.0 (1.0-1.0) | 1.0 (1.0-2.0) |  | 2.0 (1.0-3.0) | 2.0 (1.0-4.0) | 2.0 (1.0-3.0) |  | **<0.001** | 0.17 | 0.06 |
| **Peak Viral Shedding Burden** (log_10_ copies/mL), median (Q1-Q3) | 4.1 (3.7-4.5) | 4.5 (4.4-4.6) | 3.9 (3.6-4.2) |  | 4.4 (4.1-5.0) | 4.7 (4.1-5.3) | 4.3 (4.0-4.7) |  | **<0.001** | **<0.001** | **<0.001** |
| **Viral Shedding Over Time** (log_10_ copies/mL), median (Q1-Q3) |  |  |  |  |  |  |  |  |  |  |  |
| 1-day post-LAIV | 4.0 (3.6-4.4) | 4.5 (4.3-4.6) | 3.8 (3.5-4.1) |  | 4.2 (4.0-4.7) | 4.4 (4.1-5.1) | 4.2 (3.9-4.5) |  | **<0.001** | **<0.001** | **<0.001** |
| 2-4 days post-LAIV | 0.0 (0.0-3.2) | 0.0 (0.0-0.0) | 0.0 (0.0-3.4) |  | 3.7 (0.0-4.2) | 3.8 (0.0-4.5) | 3.6 (0.0-4.1) |  | **<0.001** | 0.30 | 0.09 |
| 5-7 days post-LAIV | 0.0 (0.0-0.0) | 0.0 (0.0-0.0) | 0.0 (0.0-0.0) |  | 0.0 (0.0-0.0) | 0.0 (0.0-0.0) | 0.0 (0.0-0.0) |  | **<0.001** | 0.39 | 0.16 |
| **Total Viral Shedding Burden** (log_10_ copies*days/mL), median (Q1-Q3) | 4.5 (2.2-10.0) | 4.6 (2.3-8.2) | 4.5 (2.0-10.0) |  | 12.5 (5.4-15.9) | 13.3 (6.8-17.3) | 11.2 (5.0-15.0) |  | **<0.001** | 0.27 | **0.02** |
| **Viral Clearance Profile**, n (%)^b^ |  |  |  |  |  |  |  |  | **<0.001** | 0.43 | 0.17 |
| Slow | 8 (5.0%) | 1 (1.9%) | 7 (6.5%) |  | 35 (15.7%) | 18 (19.4%) | 17 (13.0%) |  | **0.001** | 0.38 | 0.27 |
| Moderate | 33 (20.6%) | 10 (18.9%) | 23 (21.5%) |  | 107 (47.8%) | 47 (50.5%) | 60 (45.8%) |  | **<0.001** | 0.86 | 0.57 |
| Fast | 119 (74.4%) | 42 (79.2%) | 77 (72.0%) |  | 82 (36.8%) | 28 (30.1%) | 54 (41.2%) |  | **<0.001** | 0.42 | 0.19 |
|  |  |  |  |  |  |  |  |  |  |  |  |

All statistical tests comparing median values used the Mann-Whitney U Test, and all statistical tests comparing proportions used the two-proportion z-test. The chi-squared test was used to compare categorical variables.

Bold p-values represent statistical significance at the p < 0.05 level

Abbreviations: Q1, 25^th^ quartile, Q3, 75^th^ quartile, LAIV, live attenuated influenza vaccine

^a^ Proportions were calculated out of total number of individuals with detection per column

^b^ Proportions represent among those with influenza detection

**Table S5. Host and viral determinants of influenza A and B clearance**

| **Variables** | **aHR^a^ (95% CI)** | **p-value** |
| --- | --- | --- |
| **Influenza Season** |  |  |
| 2023-2024 | 0.91 (0.73, 1.15) | 0.44 |
| 2024-2025 | Ref | - |
|  |  |  |
| **Host** | | |
| **Sex** |  |  |
| Female | 0.92 (0.73, 1.16) | 0.48 |
| Male | Ref | - |
| **Age^b^** | 1.11 (0.94, 1.30) | 0.23 |
|  |  |  |
| **Virus** | | |
| **Influenza Type** |  |  |
| Influenza A | 1.62 (1.29, 2.03) | **<0.001** |
| Influenza B | Ref | - |

Abbreviations: aHR, adjusted hazard ratio, CI, confidence interval

Bold p-values represent statistical significance at the p < 0.05 level.

^a^ Multivariate Cox proportional hazards model adjusted for all other variables in table, along with adjustment for 1-day post LAIV viral load for influenza A and influenza B (both p>0.05).

^b^ Ten unit increase in age

**Table S6. Associations between host factors and viral clearance profiles stratified by influenza type**

|  | **Influenza A** | | | |  | **Influenza B** | | | |
| --- | --- | --- | --- | --- | --- | --- | --- | --- | --- |
| **Variable** | **Slow**  **(n=8)** | **Moderate**  **(n=33)** | **Fast**  **(n=119)** | **p-value** |  | **Slow**  **(n=35)** | **Moderate**  **(n=107)** | **Fast**  **(n=82)** | **p-value** |
| **Age**, Median (Q1-Q3) | 20.5  (19.0-21.8) | 22.0  (20.0-27.0) | 23.0  (20.5-27.0) | 0.14 |  | 23.0  (20.0-25.5) | 22.0  (20.0-25.5) | 23.0  (21.0-28.0) | 0.34 |
| **Sex,** n (%)^a^ |  |  |  | 0.65 |  |  |  |  | 0.08 |
| Female | 7 (87.5%) | 23 (69.7%) | 85 (71.4%) |  |  | 27 (77.1%) | 83 (77.6%) | 30 (36.6%) |  |
| Male | 1 (12.5%) | 10 (30.3%) | 34 (28.6) |  |  | 8 (22.9%) | 24 (22.4%) | 52 (63.4%) |  |
| **Influenza Vaccination**, n (%)^a^ |  |  |  | 0.26 |  |  |  |  | 0.91 |
| Yes | 6 (75.0%) | 17 (51.5%) | 78 (65.5%) |  |  | 22 (62.9%) | 69 (64.5%) | 51 (62.2%) |  |
| No | 2 (24.0%) | 16 (48.5%) | 41 (34.5%) |  |  | 13 (37.1%) | 37 (34.5%) | 31 (37.8%) |  |
| **Recent Smoking**, n (%)^a^ |  |  |  | 0.30 |  |  |  |  | 0.81 |
| Yes | 1 (12.5%) | 9 (27.2%) | 19 (16.0%) |  |  | 7 (20.0%) | 17 (15.9%) | 13 (15.9%) |  |
| No | 7 (87.5%) | 24 72.7%) | 100 (84.0%) |  |  | 28 (80.0%) | 90 (84.1%) | 69 (84.1%) |  |
|  |  |  |  |  |  |  |  |  |  |

The Kruskal Wallis Test was used to compare median values between viral clearance profiles. The Fisher exact test was used when observed count was < 5, and the Chi-squared test was used when all observed counts were > 5.

^a^ Proportions were calculated as within each viral clearance profile

**Table S7. Viral shedding decay rate is consistent within viral clearance profiles independent of influenza type or codetection status**

| **Viral Clearance Profile** | | | | | | | | |
| --- | --- | --- | --- | --- | --- | --- | --- | --- |
|  | **Slow** | |  | **Moderate** | |  | **Fast** | |
| **Variable** | **Estimate^a^** | **p-value** |  | **Estimate^a^** | **p-value** |  | **Estimate^a^** | **p-value** |
| **Days Post Vaccination** | -0.07 | 0.22 |  | -0.73 | **<0.001** |  | -2.65 | **<0.001** |
| **Influenza Type** |  |  |  |  |  |  |  |  |
| Influenza A | 0.42 | 0.15 |  | 0.25 | **0.02** |  | 0.20 | **<0.01** |
| Influenza B | Ref | --- |  | Ref | --- |  | Ref | --- |
| **Days Post Vaccination*Influenza Type** |  |  |  |  |  |  |  |  |
| Influenza A | 0.11 | 0.13 |  | -0.05 | 0.15 |  | -0.09 | 0.07 |
| Influenza B | Ref | --- |  | Ref | --- |  | Ref | --- |
|  |  |  |  |  |  |  |  |  |
| **Codetection Status** |  |  |  |  |  |  |  |  |
| Codetection | 0.31 | 0.18 |  | 0.08 | 0.52 |  | 0.22 | 0.27 |
| Single Virus | Ref | --- |  | Ref | --- |  | Ref | --- |
| **Days Post Vaccination*Codetection Status** |  |  |  |  |  |  |  |  |
| Codetection | -0.05 | 0.4 |  | 0.03 | 0.48 |  | -0.14 | 0.40 |
| Single Virus | Ref | --- |  | Ref | --- |  | Ref | --- |
|  |  |  |  |  |  |  |  |  |

Separate models for influenza type and codetection status are shown. This analysis was performed on each viral clearance profile individually. The interaction terms reported and their p-values were interpreted in the manuscript. Further details on the model methods are provided in the supplemental methods.

Bold p-values represent significance at the p < 0.05 level

^a^ Multivariate linear mixed-effects model adjusting for study year

**Table S8. Reported upper respiratory symptoms over study follow-up period and by study visit**

|  |  |  | **Study Timepoint** | | | | |
| --- | --- | --- | --- | --- | --- | --- | --- |
| **Upper Respiratory Symptom** | **Overall (n=283), No. (%)** |  | **1 Day Post-Vaccination (n=275), No. (%)** |  | **2-4 Days Post-Vaccination (n=280), No. (%)** |  | **5-7 Days Post-Vaccination (n=274), No. (%)** |
| Feeling Tired | 164 (58.0%) |  | 112 (40.7%) |  | 109 (39.6%) |  | 92 (33.5%) |
| Runny Nose | 165 (58.3%) |  | 100 (36.4%) |  | 115 (41.8%) |  | 88 (32.0%) |
| Nasal Congestion | 142 (50.2%) |  | 60 (21.8%) |  | 102 (37.1%) |  | 69 (25.1%) |
| Sneezing | 138 (48.8%) |  | 56 (20.4%) |  | 91 (33.1%) |  | 67 (24.4%) |
| Sore Throat | 79 (27.9%) |  | 27 (9.8%) |  | 49 (17.8%) |  | 37 (13.5%) |
| Cough | 72 (25.4%) |  | 34 (12.4%) |  | 38 (13.8%) |  | 39 (14.2%) |
| Scratchy Throat | 68 (24.0%) |  | 24 (8.7%) |  | 37 (13.5%) |  | 32 (11.6%) |
| Itchy Nose | 59 (20.8%) |  | 18 (6.5%) |  | 41 (14.9%) |  | 25 (9.1%) |
| Other | 46 (16.3%) |  | 27 (9.8%) |  | 17 (6.2%) |  | 10 (3.6%) |
|  |  |  |  |  |  |  |  |

**Table S9. Total upper respiratory symptom burden by influenza season**

|  |  |  | Influenza Season | | |  |  |
| --- | --- | --- | --- | --- | --- | --- | --- |
|  | Overall |  | 2023-2024 |  | 2024-2025 |  | p-value^a^ |
| Total Symptom Burden, Median (Q1-Q3) | 12.8 (5.0-30.8) |  | 12.0 (3.0-26.8) |  | 13.5 (5.5-32.2) |  | 0.42 |
|  |  |  |  |  |  |  |  |

The Mann-Whitney U Test was used to compare the median values of total symptom burden between influenza seasons.

Abbreviations: Q1, 25^th^ quartile, Q3, 75^th^ quartile

^a^ p-value represents the result test comparing influenza seasons

**Table S10. Viral determinants of respiratory symptoms by influenza type**

|  | **Influenza A** | |  | **Influenza B** | |
| --- | --- | --- | --- | --- | --- |
| **Variable** | **Estimate^a^ (95% CI)** | **p-value** |  | **Estimate^a^ (95% CI)** | **p-value** |
| **Total Viral Shedding Burden** | 0.78 (0.14, 1.43) | **0.02** |  | 0.50 (0.08, 0.92) | **0.02** |
| **Total Duration of Viral Shedding** | 3.50 (0.45, 6.54) | **0.02** |  | 1.36 (-0.51, 3.23) | 0.15 |
| **Total Peak Viral Load** | 1.05 (-4.68, 6.78) | 0.72 |  | 1.86 (-2.82, 6.54) | 0.43 |
| **Viral Clearance Profile** |  |  |  |  |  |
| Slow | 11.27 (-3.06, 16.27) | 0.22 |  | 1.51 (-8.38, 11.40) | 0.76 |
| Moderate | 6.60 (-6.79, 29.32) | 0.18 |  | 3.70 (-3.61, 11.01) | 0.32 |
| Fast | Ref | - |  | Ref | - |
|  |  |  |  |  |  |

Abbreviations: CI, confidence interval

Bold p-values represent significance at the p < 0.05 level. Linear regression models only included those with influenza A detected, and influenza B detected.

^a^ Multivariate linear regression adjusting for age, sex, influenza vaccination, and environmental allergies

**Figure S1. Distribution of RP Internal Extraction Control Ct Values.** The distribution of RNaseP Ct values did not differ by study visit. The p-value shown in the figure represents the p-value of the Kruskal-Wallis test comparing the distribution of RNaseP Ct values by study visit.

**Figure S2. Schematic illustration of Area Under the Curve (AUC).** The shaded gray area represents the integral (AUC) of either viral shedding burden or symptom burden over time, which was used to compute the total viral shedding burden or total symptom burden using the trapezoidal rule for each participant.

**Figure S3. Distribution of total symptom burden stratified by detection status.** Total symptom burden differed for those without influenza A or B detected and those with influenza A or B detected (p < 0.01). (A) Total symptom burden of individuals without influenza A or B detected. (B) Total symptom burden of individuals with influenza A or B detected.

**Figure S4. Comparative symptom burden following LAIV by influenza detection status.** Spider plots display median total symptom scores across eight upper-respiratory symptoms (scratchy throat, nasal congestion, sneezing, sore throat, runny nose, cough, feeling tired, and itchy nose). (A) Participants who ever had RT-qPCR-confirmed influenza A or influenza B RNA at any post-vaccination timepoint. (B) Participants with RT-qPCR-confirmed single virus detection (only influenza A or only influenza B). Colored lines and line types show group-level symptom patterns, and shaded polygons highlight the corresponding median total symptom profiles.

**Figure S5. Distribution of total viral shedding burden.** Total viral shedding burden differed for influenza A and B (p < 0.001). (A) Total viral shedding burden for influenza A. (B) Total viral shedding burden for influenza B.

**Supplementary Methods**

Description of Primers and Probes Used in the Detection and Quantification of Influenza Virus Genetic Material

| Virus (Target) | Primer/Probe | Sequence |
| --- | --- | --- |
| Influenza A (Matrix Gene) | F Primer | 5’-CATGGARTGGCTAAAGACAAGACC-3’ |
|  | R Primer | 5’AGGGCATTTTGGACAAAKCGTCTA-3’ |
|  | Probe | ^FAM^TGCAGTCCTCGCTCACTGGGCACG^BHQ-1^ |
| Influenza B (NS1 Protein) | F Primer | 5’-TCCTCAACTCACTCTTCGAGCG-3’ |
|  | R Primer | 5’-CGGTGCTCTTGACCAAATTGG-3’ |
|  | Probe | ^FAM^CCAATTCGAGCAGCTGAAACTGCGGTG^BHQ-1^ |

Primers and Probes adapted from Boikos et. Al^14^

Limit of Detection and Assay Validation Procedures

The limit of detection (LOD) for the influenza A and B RT-qPCR assays was determined by spiking extracted human RNA from influenza-negative nasal swabs with the plasmid standard. Briefly, a total of 20 RT-qPCR reactions for the influenza A and B assays across 4 plates were performed on human matrix spiked with the plasmid standard at the following concentrations: 10^3^, 10^2^, 10, 5, and 2.5 copies per uL. Linear regression models were fit for influenza A and B, using log_10_-transformed plasmid concentrations to predict Ct values, enabling estimation of cutoffs at 2.5 and 2 copies per uL. Data for these experiments is provided below. Samples with a Ct value greater than 33.2 (influenza A) and 34.3 (influenza B) were considered negative.

| Standard Concentration (copies/uL) | Influenza A Assay Mean Ct  (n = 20 replicates) | Influenza B Assay Mean Ct  (n = 20 replicates) |
| --- | --- | --- |
| 10^3^ | 26.13 | 25.52 |
| 10^2^ | 29.18 | 28.34 |
| 10 | 32.70 | 31.75 |
| 5 | 33.51 | 32.48 |
| 2.5 | 34.56 | 33.21 |

Influenza A and B Detection and Quantification

Each RT-qPCR assay included an in-run standard curve using 10-fold serial dilutions of a plasmid standard, ranging from 10^2^-10^8^ copies per uL. The plasmid standard contained the target sequences for the influenza A and influenza B RT-qPCR assays. Each RT-qPCR assay included a negative template control, and a negative human subject control (extracted human nasal swab RNA from influenza-negative individuals).

Linear Mixed Effects Models for Viral Load Decay

To evaluate differences in viral load decay rates by influenza type and codetection status, we fit linear mixed effects models with random intercepts to account for within-subject correlation from repeated measurements. Two separate models were fit, one assessing influenza type and the other assessing single virus vs. codetection. Fixed effects in these model included influenza type (influenza A vs. B) or codetection status (single virus vs. both viruses detected), time (days-post vaccination), and an interaction term between time and influenza type or time and codetection status. The primary parameter of interest for these models was the interaction term, which was used to assess whether the rate of viral load decay differed between influenza type or codetection status.

Model coefficients for time represent the average rate of change in viral load per day for the reference influenza type (influenza X) or the reference codetection status (X). Coefficients for influenza type or codetection status represent baseline differences at time zero (1-day post-vaccination). The interaction coefficient reflects the difference in the rate of change (slope) between influenza types or codetection status. Random intercepts were included to allow each participant to have and individual baseline viral load, thereby accounting for between-subject variability.
